# Does the Distance of the Tumor from the Surgical Margin Affect Biochemical Recurrence in Patients with Pathological Organ-Confined Prostate Cancer?

**DOI:** 10.5146/tjpath.2021.01546

**Published:** 2021-09-15

**Authors:** Ayse Ozbek, Rıdvan Ozbek, Mehmet Duvarcı, Olcay Kandemir

**Affiliations:** Department of Pathology, Dr. Abdurrahman Yurtaslan Oncology Training and Research Hospital, Ministry of Health - University of Health Sciences, Ankara, Turkey; Department of Urology, Kecioren Training and Research Hospital, Ministry of Health - University of Health Sciences, Ankara, Turkey; Dr. Abdurrahman Yurtaslan Oncology Training and Research Hospital, Ministry of Health - University of Health Sciences, Ankara, Turkey

**Keywords:** Surgical Margins, PSA, Prostate Cancer, Biochemical recurrence

## Abstract

***Objective:*** To investigate the effect of the distance between tumor and surgical margin on biochemical recurrence in patients with organ-confined prostate cancer.

***Material and Method:*** The data of 208 patients, who underwent radical prostatectomy between 2012-2018, were retrospectively analyzed. The surgical margin status of 147 pathologically organ-confined patients was categorized as positive, close (<1mm) and negative. Surgical margin status and parameters affecting biochemical recurrence were examined. Furthermore, multivariate analysis was done to determine the parameters associated with biochemical recurrence.

***Results:*** Biochemical recurrence was detected in 21 (14.2%) of 147 patients. 38 (27.9%) men had negative surgical margins, 68 (46.2%) had close surgical margins and 41 (25.9%) had positive surgical margins. Tumor volume and ISUP grade were found to be statistically significant for positive surgical margin and close surgical margin patients compared to negative surgical margin patients. Close surgical margin was not statistically associated with biochemical recurrence. Preoperative high PSA (p<0.001) and positive surgical margin (p=0.021) were independent risk factors for biochemical recurrence.

***Conclusion:*** According to our results, it is not necessary to include the presence of a close surgical margin in the pathology reports in patients with pathological organ-confined tumors and negative surgical margins.

## INTRODUCTION

Biochemical recurrence (BCR), which is one of the important markers in predicting prognosis following radical prostatectomy, is associated with various factors, and surgical margin positivity is one of the leading ones. Studies reveal that surgical margin positivity is an independent predictive factor in terms of BCR and cancer-specific mortality (1–3).

On the other hand, BCR in cases with localized prostate carcinoma, where the positive surgical margin is not observed, suggests that not only the tumor’s contact with the surgical margin but also its proximity may affect the development of recurrence (4–7).

Although positive surgical margin rates decrease with developing surgical techniques, radical prostatectomy operations performed to maintain urinary and sexual function generally cause the prostate to be removed with surgical margins close to the tumor.

Even if the tumor is too close to an inked surface, the surgical margin is considered negative if it does not come into contact with the ink. However, the definition of surgical margin proximity for prostate cancer is not yet clear. In some cancers, the tumor’s proximity to the surgical margin has been shown to be correlated with the risk of recurrence. For example, in colon carcinoma, it has been stated that tumors close to the radial surgical margin and tumors that contact with the surgical margin have a similar rate of local and distant recurrence (8,9). In prostate cancer, the cut-off values taken for the definition of the tumor’s proximity to the surgical margin vary and the effect of this factor on biochemical recurrence remains unclear.

We aimed to investigate the effect of surgical margin status on biochemical recurrence in pathologically organ-confined prostate cancer.

## MATERIALS and METHODS

This study was approved by the ethics committee (approval number 2019-04/254). The data of 208 patients, who were treated with radical prostatectomy for prostate adenocarcinoma between 2012 and 2018 in a single referral center, were retrospectively analyzed. 10 patients with node positive (pN+), 23 patients with seminal vesicle invasion (pT3b), and 28 patients with extracapsular extension (pT3a) were excluded from the study. None had preoperative androgen deprivation therapy or radiotherapy. In total, 147 patients (pT2) were enrolled in the study.

Clinical and pathological data included age, prostate specific antigen, prostate volume, tumor volume, International Society of Urological Pathology (ISUP) grade group, lymphovascular invasion, perineural invasion, biochemical recurrence and surgical margin status. Follow-up schedules were developed by the surgeon in accordance with the European Association of Urology (EAU) prostate cancer guidelines. Biochemical recurrence was defined as PSA≥0.2 ng/ml.

### Analysis of Surgical Specimens

**Figure 1:**
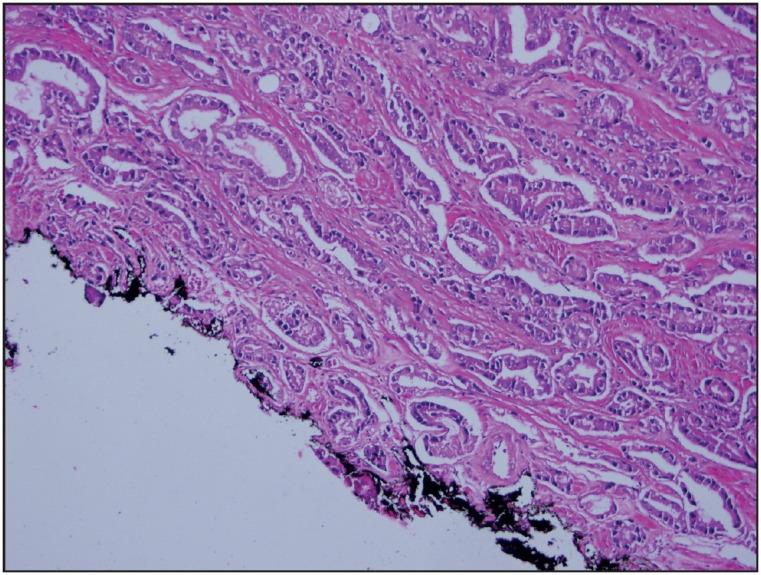
Tumor reaches the inked surgical margin (H&E, x200).

**Figure 2:**
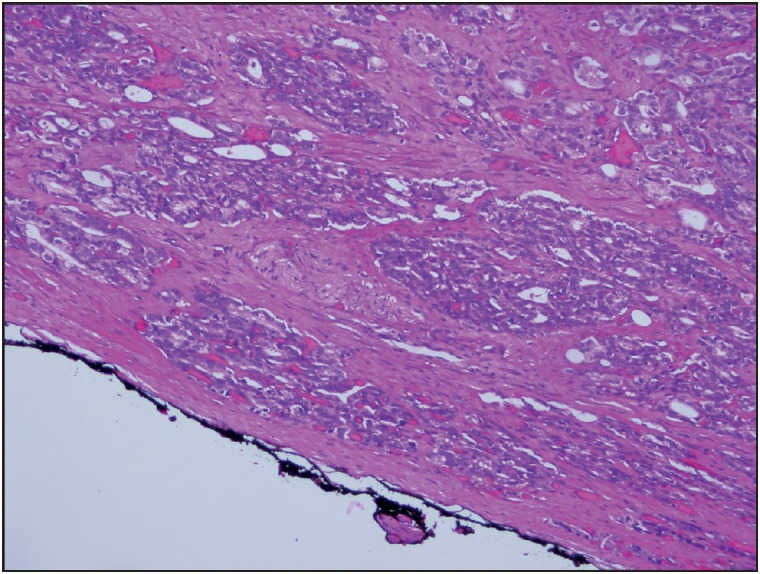
Tumor approaching to within 1 mm of the margin (H&E, x200).

Before macroscopic sampling, all specimens were left in 10% formalin for 18-24 hours. Afterward, the dimensions of the specimens were recorded, and the specimen surfaces were painted with ink. The entire material was sliced from the apical (distal) to the basal (bladder neck) into 3 mm thick sections and blocked for embedding in paraffin. Two sections with 3-4 micron thickness were taken from the blocks and examined under a microscope. All cases were re-evaluated by one pathologist according to the 2014 ISUP modified Gleason grading system. While evaluating surgical margins, contact of tumor cells with the dye in the areas with surgical border staining was considered as positive surgical margin (PSM) (Figure 1). A close surgical margin (CSM) was reported when the tumor approached the margin by less than 1 mm (Figure 2).

### Statistical Analysis

All statistical analyses were performed using the SPSS 22.0 (IBM SPSS Statistics, IBM Corporation, Chicago, IL) software for Windows. The compliance of the numeric variables with the normal distribution was assessed with the Kolmogorov-Smirnov test. Demographic and clinicopathologic features were compared between the three surgical margin groups using Kruskal-Wallis tests and One-Way Anova tests for continuous variables and Chi squared tests for categorical variables. Significant parameters in the Kruskal-Wallis test were compared in binary groups using the Mann-Whitney U test. The median (interquartile range, IQR) was used to define the non normally distributed variables, while frequency and percentage were used for categorical variables. In all the analyses, the statistical significance level was accepted as 0.05.

## RESULTS

The study group included 147 patients with pathologically organ-confined prostate cancer. Of these, 38 (27.9%) men had negative surgical margins (NSM), 68 (46.2%) had close surgical margins and 41 (25.9%) had positive surgical margins. Demographic and clinicopathologic data are presented in Table 1.

**Table 1:** Patients’ demographic and clinicopathologic characteristics by surgical margin status

	**NSM (n=38 )**	**CSM (n=68)**	**PSM (n=41)**	**Overall (n=147)**	**p**
Age, years Mean ± SD	62.6±5.23	63.08±6.16	63.0±6.07	62.95±5.87	0.943A
PSA (ng/mL) Median(IQR)	7.25 (4.99-11.25)	* 6.9 (4.92-8.22)	8 (6.21-12.49)	7 (5.3-10)	***0.021K***
Prostate volume (mL) Median(IQR)	42.5 (30.25-61)	46 (33-675)	39 (26-55.5)	43 (31-62)	0.211K
Tumor volume (mL) Median(IQR)	* 2.07 (0.76-3.77)	* 2.87 (1.63-6.51)	4.25 (1.65-10.2)	2.6 (1.53-6)	***0.021K***
ISUP Grade, n (%) 1 2 and 3 4 and 5	28 (35.4) 8 (14.8) 2 (14.3)	37 (46.8) 27 (50) 4 (28.6)	14 (17.7) 19 (35.2) 8 (57.1)	79 (53.7) 54 (36.7) 14 (9.5)	***0.004c***
PNI, n (%) Present Absent	15 (23) 23 (28)	29 (44.7) 39 (47.6)	21 (32.3) 20 (24.4)	65 (44.2) 82 (55.8)	0.370c
LVI, n (%) Present Absent	1 (20) 37 (26)	4 (80) 64 (45)	0 (0) 41 (29)	5 (3.4) 142 (96.6)	0.303c

**PSA:** Prostate-specific antigen, **ISUP:** International Society of Urological Pathology, **PNI:** Perineural invasion, **PSM:** Positive surgical margin, **NSM:** Negative surgical margin, **CSM:** Close surgical margin, **LVI:** Lymphovascular invasion, **PNI:** Perineural invasion, **IQR:** Interquartile range A: Anova (one-way), c: Chi-Square test, K: Kruskal-Wallis test (Mann-Whitney U test)/ * difference with PSM

PSA values were found to be significantly higher in the patients with PSM compared to the patients with CSM (p=0.005). Tumor volume was statistically significantly higher in PSM and CSM groups compared to NSM (p=0.018 PSM vs NSM, p=0.007 CSM vs NSM). Furthermore, it was observed that the patients in the PSM and CSM groups had higher ISUP grade compared to the patients in the NSM group (p=0.004). After a median follow-up of 12.3 months, BCR occurred in 4 (19%), 5 (23.9%), and 12 (57.1%) patients with negative, close and positive surgical margins, respectively (Table 2).

**Table 2:** Recurrence rates with surgical margin status

**Biochemical recurrence**	**Margin status**			**Total**
	**Negative**	**Close**	**Positive**	
Present, n (%)	4 (19)	5 (23.9)	12 (57.1)	**21**
Absent, n (%)	34 (27)	63 (50)	29 (23)	**126**
Total, n (%)	**38 (27.9)**	**68 (46.2)**	**41 (25.9)**	**147 (100)**

*Median follow-up: 12.3 months*

In univariate analysis, we discovered a statistically significant relationship between preoperative PSA value (p<0.001), tumor volume (p=0.001), ISUP grade group 4-5 (p=0.027), PSM (p=0.017), CSM (p=0.006) and BCR. In multivariate analysis, a high preoperative PSA value (p<0.001) and PSM (p=0.021) were found to be statistically significant (Table 3).

**Table 3:** Uni- and multivariate logistic regression analysis for predictors of Biochemical Recurrence.

	**Univariate analysis**			**Multivariate analysis**		
	**OR**	**95% CI**	**p**	**OR**	**95% CI**	**p**
Age	1.011	0.939-1.089	0.764			
PSA (ng/mL)	1.333	0.171-1.517	***<0.001***	1.312	1.157-1.489	***<0.001***
Prostate volume (mL)	1.010	0.997-1.024	0.125			
Tumor volume (mL)	1.144	1.058-1.237	***0.001***			
ISUP Grade group 4-5	3.537	1.151-10.871	***0.027***			
PSM	4.127	1.745-9.759	***0.001***	3.441	1.204-9.837	***0.021***
CSM	0.234	0.083-0.664	***0.006***			
NSM	1.443	0.566-3.679	0.442			

**OR:** Odds ratio, **CI:** Confidence interval, **PSA:** Prostate specific antigen, **ISUP:** Internatıonal Society of Urological Pathology, **PSM:** Positive surgical margin, **CSM:** Close surgical margin, **NSM:** Negative surgical margin.

## DISCUSSION

European Association of Urology (EAU) current guidelines state that surgical margin positivity, high preoperative PSA, high ISUP grade and high pathological stage in the specimen after radical prostatectomy are risk factors in terms of BCR (10). In our study, the relationship between the distance of the tumor to the surgical margin and BCR was investigated in the group with pathological organ-confined prostate cancer (pT2). As a result of the multivariate analysis, it was determined that the distance of the tumor to the surgical margin did not affect the BCR, while the independent factors affecting the BCR were surgical margin positivity and preoperative PSA value.

Biochemical recurrence may develop during follow-up in the patients who had organ-confined prostate cancer and underwent radical prostatectomy. In a recent study conducted by Stolzenbach et al., 5-year BCR-free survival rates in pathological organ-confined prostate cancer were reported to be approximately 88% (11). Aoun et al. associated the BCR development in pT2 prostate cancer with factors such as insufficient diagnosis of surgical margin positivity, limited sections examined even though the entire specimen was sampled, PSA secretion of the postoperative residual benign prostate tissues and occult lymph node metastasis (12).

The presence of tumor closer than 1 mm to the painted surgical margin was accepted as CSM when defining the proximity to the surgical margin in this study. While determining this, we considered the existence of the publications accepting a value of 1 mm in the literature (13,14). Whalen et al. have found that BCR developed more commonly in patients with close surgical margins than patients with negative surgical margins. They even stated that the patients with CSM showed similar rates of BCR development as the patients with PSM (13). Similarly, Herforth et al. have shown that proximity to the surgical margin increased BCR. However, they emphasized that in the absence of high-risk parameters for BCR, surgical margin status alone did not increase metastasis, prostate cancer specific mortality and all-cause mortality, and that surgical margin status alone should not be used to decide adjuvant treatment (14). In our study, contrary to these studies, we found that the presence of tumor close to the surgical margin did not affect BCR in organ-confined disease.

In some studies, the presence of a tumor closer than 0.1 mm to the surgical margin was accepted as ‘close surgical margin’. Lu et al. have found that the Gleason score and the presence of positive surgical margin were the strongest prognostic factors affecting BCR, and the presence of tumors close to the surgical margin led to the development of BCR (15). In another study, Izard et al. have shown that the presence of tumors close to the surgical margin had a similar rate of BCR risk as in the patients with positive surgical margins. In these studies, the distance of 0.1 mm was measured by comparing it to either the visual field diameter or fibroblast size (16). We think that the evaluation of this proximity according to the visual field diameter or the width of a few fibroblasts is subjective and will vary between pathologists. Moreover, prostatectomy materials are macroscopically sampled in approximately 3 mm thick pieces and then 3-4 micron sections are obtained and examined. The fact that the tumor is closer than 0.1 mm in these sections suggests that the surgical margin may be positive in the following sections as stated by Izard et al. in their study, and this situation remains uncertain since all tissues cannot be examined microscopically. For these reasons, we considered the presence of a tumor closer than 1 mm to the surgical margin as ‘close surgical margin’ in our study.

EAU prostate cancer guidelines have emphasized that extraprostatic spread, seminal vesicle invasion and lymph node invasion may increase the risk of BCR (10). In the literature, there are patients with these parameters in the study cohorts that indicate a relationship between the distance to the surgical margin and BCR. We believe that evaluating these parameters and proximity to the surgical margin together affects the research results. Therefore, we have excluded the patients with extraprostatic invasion, seminal vesicle invasion and lymph node positivity on pathology in the study group.

This study has some limitations to consider, including the retrospective design and shorter follow-up time compared to other studies. In addition, the relatively small number of the patients is seen as another limiting factor. The reason for this is that patients with organ-confined prostate cancer (pT2) were included in the study. Although this limits the number of patients, it makes our study more valuable. Moreover, the evaluation of all sections by the same pathologist eliminated an interobserver evaluation difference.

## CONCLUSION

In our study, we showed that preoperative PSA value and PSM are independent factors that increase the risk of BCR in the multivariate analysis in patients with organ-confined prostate cancer. We also found that tumor closer than 1 mm to the surgical margin had no effect on the risk of BCR. In the light of these results, we believe that the presence of a close surgical margin should not be included in the pathology reports of patients with pathological organ-confined and negative surgical margin.

## Conflict of INTEREST

The authors of this manuscript declare no conflict of interest.

## FUNDING

The authors state that this work has not received any funding.
